# A dataset of color QR codes generated using back-compatible and random colorization algorithms exposed to different illumination-capture channel conditions

**DOI:** 10.1016/j.dib.2022.108780

**Published:** 2022-11-24

**Authors:** Ismael Benito-Altamirano, David Martínez-Carpena, Olga Casals, Cristian Fàbrega, Andreas Waag, Joan Daniel Prades

**Affiliations:** a MIND/IN2UB, Department of Electronic and Biomedical Engineering, Universitat de Barcelona, Carrer de Martí i Franquès, 1, Barcelona, 08028, Barcelona, Spain; b ColorSensing SL, Carrer Morales, 21, 1L, Barcelona, 08029, Barcelona, Spain; c Institute for Semiconductor Technology, Braunschweig University of Technology, Universitätspl. 2, Braunschweig, 38106, Lower Saxony, Germany; d Department of Mathematics and Computer Science, Universitat de Barcelona, Gran Via de les Corts Catalanes, 585, Barcelona, 08007, Barcelona, Spain

**Keywords:** Barcodes, QR Codes, Color correction, Color calibration, Colorchecker, Colorimetry

## Abstract

Color QR Codes are often generated to encode digital information, but one also could use colors or to allocate colors in a QR Code to act as a color calibration chart. In this dataset, we present several thousand QR Codes images generated with two different colorization algorithms (random and back-compatible) and several tuning variables in these color encoding. The QR Codes were also exposed to three different channel conditions (empty, augmentation and real-life). Also, we derive the SNR and BER computations for these QR Code in comparison with their black and white versions. Finally, we also show if ZBar, a commercial QR Code scanner, is able to read them.

**Specifications Table**

**Table:** 

Subject	Computer Vision and Pattern Recognition
Specific subject area	Machine-readable patterns for colorimetry applications
Type of data	Image, Table
How the data were acquired	Initially, the QR Codes were generated using a Python code based on a modified library to create QR Codes. The QR Codes were generated using different amounts of colors and different methods of embedding. Once these QR Codes were generated, they were exposed to different channel conditions: an empty channel, an augmentation channel and a real-life channel (using a capture station). After these, for each QR Code generated in such fashion, a computation was made of the SNR and BER figures in comparison to the same QR Code without colors. Finally, we present the results of readability of those QR Codes using a commercial QR Code scanner, ZBar.
Data format	Raw, Analyzed
Description of data collection	QR Codes were generated using different: • *QR Code version*, from 5 to 9; • *color substitution ratio*, from 1 to 100 %; • *color zone*, data, error correction and both of them; • *color method*, random or back-compatible; After that, the QR Codes were exposed to three different channel conditions (empty, augmented and real life).
Data source location	• Institution: Universitat de Barcelona • City/Town/Region: Barcelona, Catalonia • Country: Spain • Latitude and longitude: 41.38, 2.11
Data accessibility	Repository name: Mendeley Data Data identification number: 10.17632/35kj4v96cm.2 Direct URL to data: https://data.mendeley.com/datasets/35kj4v96cm/2 Instructions for accessing these data: Download the data from Mendeley data repository in TAR.GZ or ZIP formats.
Related research article	Ismael Benito-Altamirano, David Martínez-Carpena, Olga Casals, Cristian Fàbrega, Andreas Waag, Joan Daniel Prades, **Back-compatible Color QR Codes for colorimetric applications,** Pattern Recognition

## Value of the Data

• There is no previous dataset of QR Codes with colors embedded using random and back-compatible algorithms.
• This dataset allows comparing different methods (i.e. color substation ratio, color zones, …) of embedding colors in QR Codes.
• At the same time, there are no other datasets of QR Codes with similar different illumination-capture channel conditions.
• This dataset provides diverse examples of QR Codes with varying conditions, which can be impactful as training data of machine-learning algorithms.
• The data can be used as raw images of QR Codes with diverse conditions, i.e. experiments to test how resilient QR Codes are under different illumination conditions in terms of error correction blocks.
• The data can be used to compare how different methods of embedding colors in QR Codes affect the barcode readability in terms of bit error ratio and signal-to-noise ratio.

## 1 Objectives

The presented dataset is part of published reasearch where we present a new methodology to embed colors in QR Codes in a back-compatible approach, this approach enables us to use QR Codes as colorn rendition charts, thus providing both colorimetric references in captured scenes and the barcode digital information. This way, linking the colorimetry problem to a standard barcode solution enables treaceability towards the color measurement. This construction method *de facto* constitutes a proposal of a new computer vision pattern, a so-called Back-Compatible Color QR Code [1].

Here we present the dataset ifself and how it was created to evaluate the impact of color inside QR Codes, i.e.how color affects: the QR Code zones, the error level, etc. This dataset is useful for any related work with barcodes and how efficiently colors can be embedded on them without affecting the readability of the barcode itself.

## 2 Data Description

The data is organized in folders. Each folder contains a generation of QR Codes we created, we created a total of 3 batch generations. Table 1 contains a summary of each batch created. A detailed explanation of the experimental variables is provided below. Batch 1 and 2 were exposed to an empty channel and an augmentation channel, batch 3 was a reduced batch that was really printed and exposed to a colorimetric setup in our laboratory facilities.

**Table 1:** Summary of parameter values for each batch generated. All batches share common parameters, at least each batch has 72 different QR Codes were generated using as reference the multiplication of the shared parameters.

All batches	Values	Size
Color substitution (%)	1, 5, 10, 15, 20, 30, 40, 50, 60, 70, 80, 100	12
Colorized zone	EC, D, EC&D	3
Colorizing method	Random, Grayscale	2
Batch 1	Values	Size
Digital IDs	from 000 to 999	1,000
QR version	5, 6, 7, 8, 9	5
Channels	Empty, Image augmentation	1 + 1
Batch 2	Values	Size
Digital IDs	000	1
QR version	5, 6, 7, 8, 9	5
Channels	Empty, Image augmentation	1 + 1,000
Batch 3	Values	Size
Digital IDs	000	1
QR version	5	1
Channels	Empty, Colorimetry setup	1 + 25

Each folder then contains a subfolder tree with the following folders: “qr”, “channel_1”, “channel_2”. First, “qr” contains the QR Code images without any color. Then, “channel_1” is the empty channel it contains all the colored images of the QR Codes and the metrics (SNR, BER and readability) of the empty channel (only taking into account color embedding). Finally, “channel_2” contains the images of the QR Codes after being exposed to an augmentation channel for batch 1 and 2, and a real-life printing-capturing process for batch 3. Table 2 show the total amount of image for these folders for each batch.

**Table 2:** Total number of images classified by batch, type of barcode and channel. The first half of the table shows the number of classic QR Codes and QR Codes with embedded colors at each batch. The second half, shows how many images are obtained from the previous Color QR Codes using the selected channels. As it is shown in Table 1, each batch uses only two of the three different channels.

**Total by barcode**	Batch 1	Batch 2	Batch 3
QR Codes	5000	5	1
Color QR Codes	360,000	360	72
**Total by channel**	Batch 1	Batch 2	Batch 3
Empty	360,000	360	72
Image augmentation	360,000	360,000	-
Colorimetry setup	-	-	1,800

Each image of a QR Code follows a simple structure, if the QR Code has no colors it follows:

**Table:** 

Name structure:
qr_$id_$version.png
Example:
qr_000_5.png

When color is encoded, this structured is enhanced:

**Table:** 

Name structure:
qr_$id_$version_$substitution_$zone_$method.png
Example:
qr_000_5_100_error-correction_random.png

For each image in “channel_0” and “channel_1” folders, we have attached a JSON file containing the derived metrics comparing the image itself with its equivalent without color embedding, in the folder “qr”.

## 3 Experimental Design, Materials and Methods

The three different batches of images in this dataset were generated with the same initial steps, only differing in the last one. First, we used a Python code based on a modified library to create QR Codes [2,3] with the desired parameters (QR Code version, digital ID and error correction level). Then, we chose a palette of colors to insert, which must be as random as possible in terms of distribution along the RGB cube. Once the palette is chosen, we embedded it in each QR Code, with two different methods of insertion: using a random insertion that avoids the computer vision patterns, and using the back-compatible proposal explained in the companion article. Finally, we simulated three different channels with noise, presenting alterations to each QR Code image. At each batch, we used different parameters and combination of channels, as explained in Table 1.

### 3.1 QR Code Versions and Digital IDs

The encoded data and the version of the QR Code will shape the actual geometry of the barcode, thus it will determine the original image pixels. To generate the barcodes, we choose as payload data a URL with a unique identifier such as https://color-sensing.com/#000, where the numbers after ‘#’ range from 000 to 999 to make the barcodes different from each other.

**Fig. 1:**
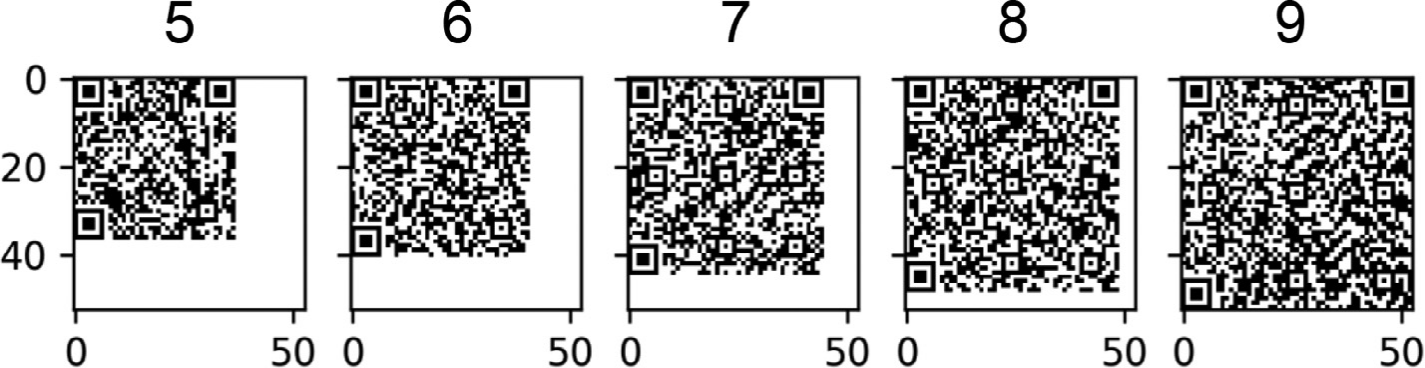
Comparison between QR Code versions, from left to right versions 5, 6, 7, 8 and 9, all containing the same string “https://color-sensing.com/#000”. Each version increase adds four rows and four columns to the previous matrix, from 37 × 37 pixels (version 5) to 53 × 53 pixels (version 9).

On the other hand, QR Codes can take several increasing versions, from v1 to v40. Each time the version is increased, these barcodes increase its size and data capacity when version increases [3] (see Fig. 1). The QR Code selected versions ranged from 5 to 9, to test and exemplify the most relevant computer vision pattern variations defined in the QR Code standard. For all of these barcodes, we used the highest level of error correction of the QR Code standard: the H level, which provides a 30% of error correction.

### 3.2 Color Generation and Substitution

**Fig. 2:**
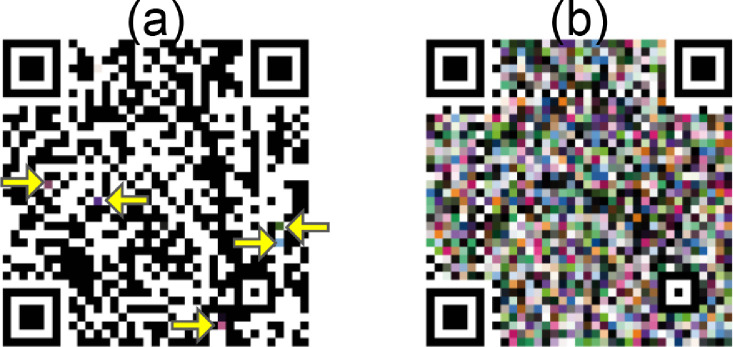
The same QR Code with data https://color-sensing.com/#000 is populated with different amounts of colors. (a) 1% of the pixels are substituted using a random placement method (yellow arrows show the colorized pixels). (b) 100% of the pixels are substituted using a random placement method.

During the generation of the batches, we filled QR Codes with a palette of random colors. The color substitution factor ranged from only 1% of the available pixel positions in a QR Code replaced with colors up to 100% (see Fig. 2). We consider only pixel positions outside the patterns used in the computer vision process of finding and aligning the QR Code. Evidently, each QR Code version offers different numbers of pixels and thus positions available for color substitution.

**Fig. 3:**
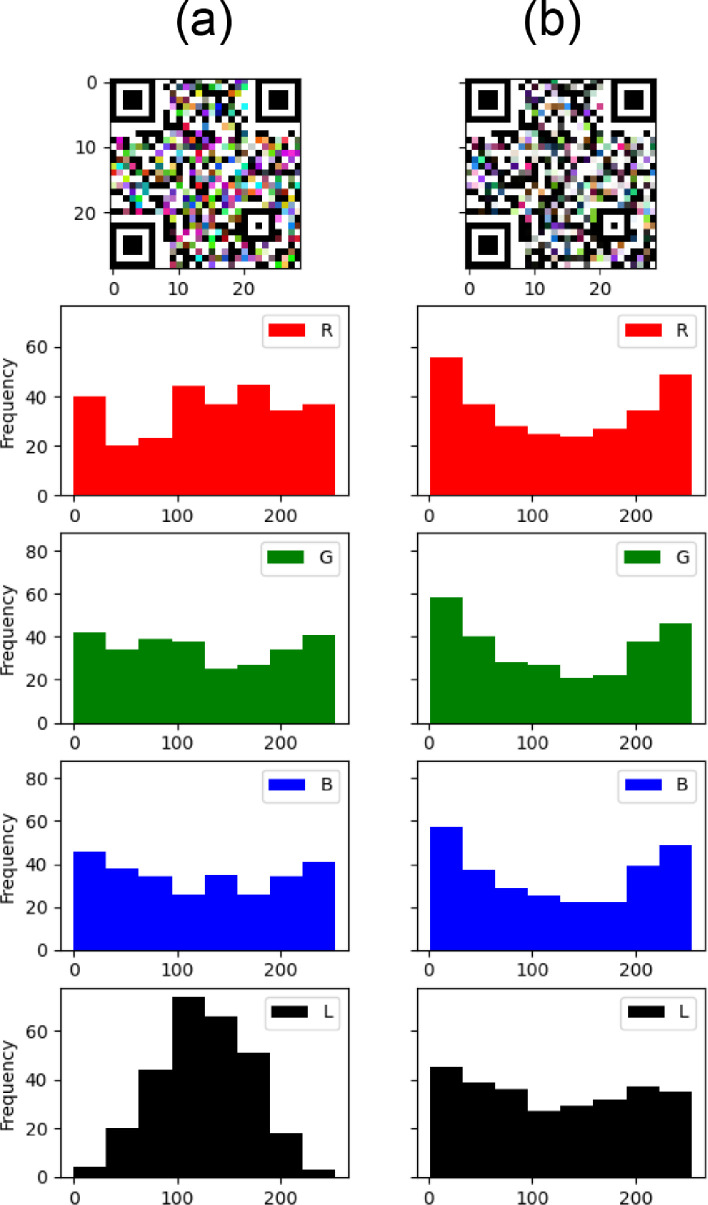
Histogram comparison between uniform randomly generated RGB channels. (a) which yields to a non-uniform grayscale -L- and uniform randomly generated grayscale -L-. (b) with derived pseudo-uniform RGB channels.

We chose our random color palette for the experiments to be representative of the RGB space. Nevertheless, the palette should be random in a way that it is uniformly random in the grayscale space L. But if we defined three uniform random RGB channels as our generator, we will have failed to accomplish a grayscale uniform random channel (see Fig. 3.a). This is due to the fact that when computing the L space as a mean of the RGB channels, we are creating a so-called Irwin-Hall uniform sum distribution [4]. In order to avoid this, we propose to first generate the L channel as a uniform random variable, then generate RGB channels which produces these generated L channel values (see Fig. 3.b).

### 3.3 Placing Colors Inside the QR Code

**Fig. 4:**
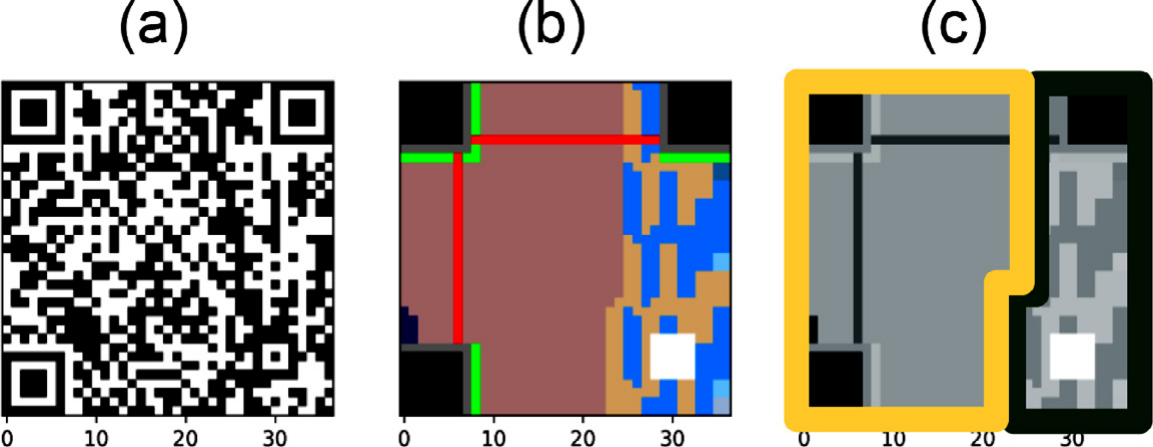
QR Code encoding defines a complex layout with several patterns to be considered, some of them are non-variant patterns found in each QR Code, others may appear depending on the size of the QR Code, and area related to the data changes for each encoding process. (a) A QR Code with high error correction level and version 5, encoding the string “https://color-sensing.com/#000”. (b) The pattern structure of (a) finder (black), alignment (white), quiet (gray) and timing (red) are invariant to the data encoded in the QR Code; non-used (dark blue), version (green) and alignment (white) depend exclusively on the version of the QR Code; data (light blue), padding (brown), error correction (dark brown) depend on the error correction level selected and the data encoded. (c) Simplified view of the QR patterns, yellow frame corresponds to the “error correction” area and dark green frame corresponds to the “data” area.

When placing colors in a QR Code, a totally random placing can break the readability assumptions of its patterns. In particular, an interested problem is studying in which zones of the QR Code we can embed colors while preserving the readability of its encoded data. The QR Code standard defines two main zones (see Fig. 4): a collection of patterns used in the reading and alignment procedure, and the main zone, used for data and error correcting information about the data. In particular, a QR Code can become unreadable after modification of a few pixels in the pattern's zone. For this reason, we restricted all insertion of colors to the main zone of data and error correcting information.

**Fig. 5:**
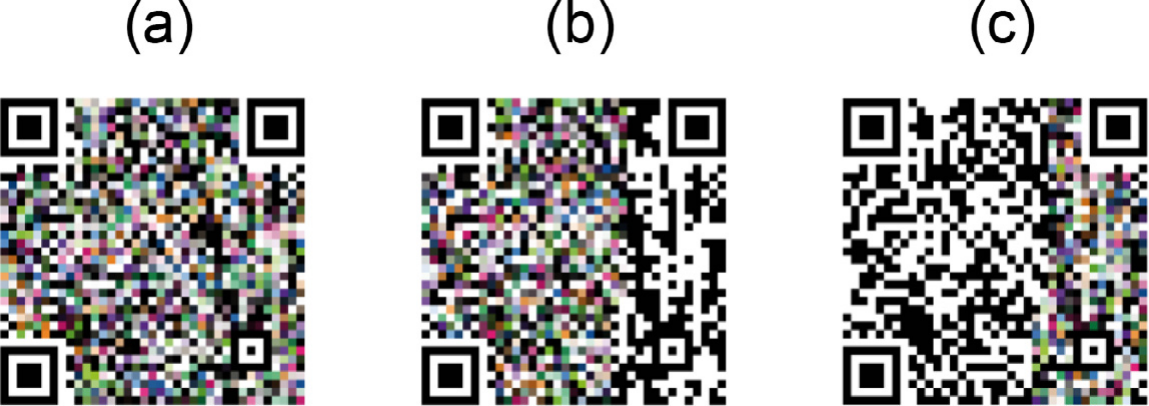
The same QR Code with data https://color-sensing.com/#000 is populated in different areas with 80% of colors for each area. (a) The whole QR Code is populated (EC&D). (b) Only the error correction area is populated (EC). (c) Only the data area is populated.

Once we have chosen to avoid the patterns and key protected areas, we are left with only two regions to use: the data and the error correction. Part of the dataset is generated by exploring all possible combinations of these two regions (see Fig. 5):

I. **EC&D.** Exclude only the pattern's zone, and allow covering with colors all the error correction and data regions (see Fig. 5.a),
II. **EC.** Only allows embedding colors in the error correction region (see Fig. 5.b),
III. **D.** Only allows embedding colors in the data region (see Fig. 5.c).

The next natural problem to ask is how we decide in what pixel we insert each color in the palette. To this end, we considered two methods of insertion:

I. **Random.** The simple method of insertion, which only selects as many free pixels as needed for the palette using random choices inside the selected region.
II. **Grayscale.** The back-compatibility proposal presented in the companion article. This method reduces the total amount of noise and miss-classifications introduced in the QR Code when encoding colors, by taking into consideration the affinity of the colors in the palette to the black and white pixels of the QR Code (i.e. to which color it resembles the most). The name comes from the grayscale conversion applied to the palette for comparing colors to black and white pixels.

### 3.4 Channels

The use of QR Code in real world conditions imply additional sources of error, like differences in printing, different placements, ambient light effects, effects of the camera and data processing, etc. All these factors can be regarded as sources of noise in a transmission channel.

**Fig. 6:**
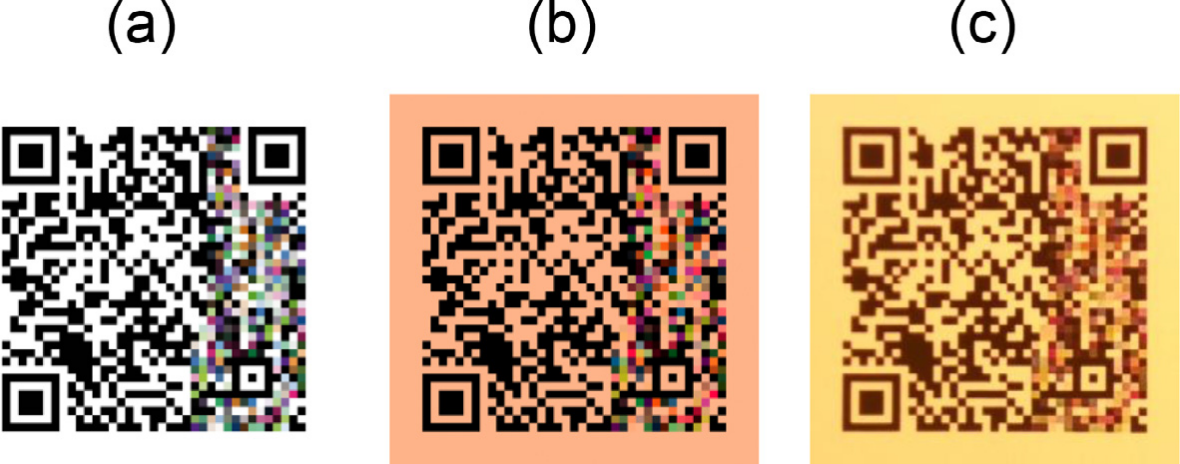
The same QR Code with data and the same amount of colors (80% of the data area) is exposed to different channels. (a) The image passed-through an empty channel. (b) The image passed-through an augmentation channel which resembles a warm light scene. (c) The image passed-through a real environment channel, actually printed and captured in a scene with a lamp at 2500K (warm light).
II. **Image augmentation.** With a data augmentation library [5], we generated images that mimic different printing processes and exposure to different light conditions. With this tool we also applied Gaussian blur distortions, crosstalk interferences between the RGB channels and changed contrast conditions. (see Fig. 6.b).
III. **Colorimetry setup.** We actually printed the QR Codes and captured them with a fixed camera (Raspberry Pi 3 with a Raspberry Pi Camera v2) [6] under different illumination-controlled conditions (Philips Hue Light strip) [7]. The camera was configured to take consistent images. The light strip was configured to change its illumination conditions with two subsets of illumination conditions: white light (9 color temperatures from 2500K to 6500K) and colored light (15 different colors sampling evenly the CIExyY space) (see Fig. 6.c).

As part of the generation of the dataset, we wanted to simulate the channel noise obtained in real world conditions. To this end, we considered 3 different channels:

I. **Empty.** A channel where there is no color alteration due to the channel. It was used as a reference, to measure the noise level induced by the colorization process (see Fig. 6.a).

Combinations of these three channels were used to generate the final images of each batch. In particular, we used an empty channel at each batch, which allows us to study the original Color QR Code without noise, and compute metrics like SNR, BER and readability. Then, at each batch we selected one of the other two channels: the first two we used image augmentation, and in the third the colorimetry setup.

### 3.5 Metrics

The differences between the original QR Code and the one obtained after a real-world capture can be regarded as noise over the visual data communication channel (printing problems, light conditions, computer vision errors, etc.). Then, this difference can be studied by using well-known noise metrics like the *signal-to-noise ratio* (SNR) and the *bit error ratio* (BER) [8]. For each generated image, the analysis of the dataset is done by computing these two metrics, and testing if a commercial QR Code scanner, ZBar [9], is able to read the data successfully.

## Ethics Statements

None of our work involved human subjects, nor animal experimentation or data collected in social media platforms.

## CRediT authorship contribution statement

**Ismael Benito-Altamirano:** Investigation, Data curation, Writing – original draft. **David Martínez-Carpena:** Software, Validation, Visualization. **Olga Casals:** Methodology, Writing – review & editing. **Cristian Fàbrega:** Resources, Project administration. **Andreas Waag:** Writing – review & editing. **Joan Daniel Prades:** Supervision, Funding acquisition.

## Declaration of Competing Interest

The research leading to this work has been developed in the research group of Prof. Joan Daniel Prades at the Universitat de Barcelona and funded by the public research project acknowledged in the paper. The resulting technology has been patented in part and is the technological core of ColorSensing, a spin-off company of the Universitat de Barcelona.

Ismael Benito-Altamirano, Olga Casals, Cristian Fàbrega, Andreas Waag and Joan Daniel Prades are coinventors of the patent “Colour correction” (PCT: WO2019145390A1), which discloses part of the preliminary ideas that underpin the research presented here. Joan Daniel Prades has stock ownership of ColorSensing.

## Data Availability

A dataset of color QR Codes generated using back-compatible and random colorization algorithms exposed to different illumination-capture channel conditions (Original data) (Mendeley Data).
